# Surgical Cases Treated at the Buffalo General Hospital

**Published:** 1871-02

**Authors:** C. C. F. Gay


ART. [illegible].—*Surgical Cases treated at the Buffalo General Hospital* By C. C. F. Gay, M. D.

*Removal of Mammary Glands for Scirrhus Disease*—Mrs. T., aged 62 years, has scirrhus of left breast—the whole gland is involved. She suffers much from the pain of it; is anxious to have it removed, although told that the prognosis is unfavorable, and that it will be likely to return after removal at no very distant period. Chloroform was administered, and I removed the entire gland, leaving only just sufficient integumental covering to coaptate the edges of the wound. The axillary glands were involved; a tumor half the size of a hen's egg, in the axilla, was not removed, but afterwards disappeared. The integuments were drawn together and secured by silver sutures. The patient convalesced rapidly; one half the wound healed by first intention, but the other half continued to suppurate; and, at one point, the disease gave evidence of developing. The disease will undoubtedly return. She left the hospital, and the result has not been ascertained.

Another case of much interest terminated as favorably as the most sanguine could hope or expect. Mrs. B., tumor of right breast. After its removal its weight was found to be three pounds. The tumor involved the entire gland; had been eighteen years growing; had increased rapidly in size during the past few weeks. The age of this patient was forty-five years. She has recently ceased to menstruate, and coincident with her climacteric period was the activity in growth of the tumor.

The tumor had, to the touch, none of the characteristics of scirrhus, neither was there pain, but the size of it made it very cumbersome; and she came from Pennsylvania, and entered the hospital with her mind made up to have it removed.

In presence of members of the hospital staff and others, I removed the entire breast; but slight hemorrhage attended the operation. The tumor was found, on examination of its inner surface, to be in part composed of scirrhus. The wound healed, in its first intention. The patient was setting up and walking about in ten days, and in thirteen days left the hospital for home, well.

*Operation for Radical Cure of Hernia.*—Two operations were made, the last of which was unsatisfactory. The patient got up too soon; the intestine partially protruded, but never to the extent of the original protrusion, and when last seen the case gave promise of ultimate cure. The first operation was a complete success. The patient, when last seen, looked as though he never had hernia; and what strikes me as a most remarkable feature of the case, is the fact of both the mental and physical improvement of the patient. He had "fits;" was somewhat mentally imbecile. Since the cure of his hernia his mind is more active; his epileptiform convulsions have disappeared, and his bodily health is much improved. The case was one of scrotal hernia. The method of operating was original; the columns of the ring were brought together by silver wire used subcutaneously, and the silk ligature inserted in order to excite sufficient local inflammation to agglutinate the parts together and prevent the ring from reappearing. I have, for a long time, thought the surgeon might and should relieve surgery of some measure of its opprobrium, by devising a method for the partial, if not the radical, cure of hernia. This ailment is certainly common enough, and the dangers attending strangulation are apparent enough to appeal to the ingenuity and dexterity of the surgeon for its effectual and permanent cure.

The truss is not a very comfortable appliance at best; and hernia, while usually supported by that truss, will, in unguarded moments, often become strangulated, requiring operation. From considerations such as these, and in view of the constant advance made in surgical science and art, it will not, I think, be unreasonable to predict, that the time is approaching when inguinal hernia will become amenable to cure—usually denominated, radical. To this end the case above is reported and given to the medical public, with the hope, also, that the method proposed for radical cure of hernia may be tried by others and found successful, as in this instance.

*Necrosis.*—A case of necrosed femur, requiring an operation of considerable magnitude, terminated favorably, although erysipelas at one time threatened to destroy the life of the patient.

One case necessitated amputation of the big toe through the continuity of the metatarsal bone. Previous operation had been made for removal of necrosed bone, with only partial relief. Pain had been constant for a long time, and the foot could not be used for purposes of locomotion. The entire foot was inflamed, and the leg swollen. The patient, therefore, very readily assented to the removal of the toe. An incision was made along the dorsum of the foot, commencing at the internal cuneiform bone and terminating at the intersection of the large toe with the toe adjoining; another incision at an acute angle with the former extended around over the other side of the toe, and terminating at the same place with the former; the integuments were then dissected back to the form flaps, and the metatarsal bone sawn through in a direction quite oblique. Two vessels required ligation. The flaps were secured by silver suture, and union was secured by first intention. The stump presented a handsome appearance, and the shoe can be worn with much more comfort, with this form of stump, than with the abrupt stump which would follow amputation at the metatarso-phalangeal articulation. The inflammation and pain of the foot at once disappeared, and the patient was able to use the foot in walking in ten days after the operation.

